# Association between exposure to proton pump inhibitors and hyperuricemia: risk prediction model establishment and nomogram construction

**DOI:** 10.3389/fphar.2026.1842574

**Published:** 2026-08-07

**Authors:** Rong-Zhong Wang, Si-Qin Sun, Ming-Xing Mao, Jia-Li Peng, Jing-Shu Dong, Xiao-Fang Xiong, Si-jia Liu

**Affiliations:** 1 West China Hospital, Sichuan University, Chengdu, China; 2 Reproductive and Maternal and Child Hospital, Chengdu University of Traditional Chinese Medicine, Chengdu, China

**Keywords:** hyperuricemia, nomogram model, PPI, retrospective study, risk prediction

## Abstract

**Purpose:**

This study aims to explore whether the exposure to proton pump inhibitor (PPI) is a potential independent risk factor for the development of new-onset hyperuricemia in patients who were hospitalized due to gastrointestinal bleeding, and to establish a validated clinical prediction indicator.

**Methods:**

We conducted a retrospective cohort study involving inpatients of the gastroenterology department admitted to West China Hospital between August 2022 and August 2025. LASSO and logistic regression were utilized to determine potential risk factors, and a nomogram prediction model was subsequently established accordingly. The model’s discriminative ability was assessed using the area under the receiver operating characteristic (ROC) curve (AUC). Calibration curves and decision curve analysis (DCA) were utilized to evaluate the model’s accuracy and clinical utility.

**Results:**

Of the 1900 patients enrolled, 10.5% developed new-onset hyperuricemia. Multivariate analysis identified PPI as a significant independent risk factor for new-onset hyperuricemia with adjusted odds ratio (OR) (95% CI) of 4.86 (3.04, 7.78). The nomogram incorporated ten predictors: gender, age, hospital time, BMI, smoking, triglyceride levels, PPIs, CCBs, diuretics, and hyperlipidemia. The model exhibited good discrimination, with AUCs of 0.759 (95% CI: 0.718∼0.800) and 0.727 (95% CI: 0.654∼0.799) in the training and validation set, respectively. The Hosmer-Lemeshow test demonstrated the model was well-calibrated (*P* > 0.05), and DCA confirmed that the model provided significant clinical net benefits.

**Conclusion:**

PPI exposure may be a potential independent risk factor for new-onset hyperuricemia in patients who were hospitalized due to gastrointestinal bleeding. The developed nomogram demonstrates good predictive performance and may assist clinicians in assessing hyperuricemia risk and optimizing individualized patient management.

## 1 Introduction

Proton pump inhibitors (PPIs) are potent acid-suppressive agents that specifically target the H^+^/K^+^-ATPase of parietal cells in the gastric fundic glands (Wang et al., 2024). They are widely used in clinical practice to manage acid-related disorders, including gastroesophageal reflux disease and peptic ulcer disease (Clarke et al., 2022; Savarino et al., 2018). Additionally, PPIs serve as first-line therapy for preventing stress-related mucosal lesions and eradicating *Helicobacter pylori* infection (Shin and Kim, 2013). Despite their well-established efficacy, the safety profile of long-term or inappropriate PPI use has attracted growing concern. Common adverse effects of PPIs include hypersensitivity reactions and gastrointestinal dysfunction. Furthermore, accumulating evidence indicates that prolonged PPI administration is positively correlated with an increased risk of stroke, lower respiratory tract infections, Parkinson’s disease, and depressive disorders (Salvo et al., 2021; Xia et al., 2024; Moayyedi and Leontiadis, 2012). In recent years, epidemiological studies have found an association between PPI exposure and increased risks of biliary tract cancer, rheumatoid arthritis, type 2 diabetes mellitus, inflammatory bowel disease, and cholelithiasis (Xia et al., 2021; Yang et al., 2021).

The association between PPIs and gout has emerged as a research focus in recent years. Kraus et al. (Kraus and Flores-Suarez, 1995) initially reported two cases of patients with duodenal bulb ulcer who developed acute gout after omeprazole treatment. Subsequent cohort study (Zhu et al., 2023) has shown that PPI use is independently associated with an increased risk of gout, with a more significant correlation within the first 30 days of medication initiation. Xu (2024) further confirmed a positive correlation between the two. Additionally, PPI dosage was associated with prolonged hospital stay, suggesting the need for caution regarding excessive use. The underlying mechanism may be that PPIs block H^+^/K^+^-ATPase, leading to the accumulation of serum adenosine triphosphate (ATP). ATP is then metabolized into adenine and ultimately converted into uric acid, which elevates serum uric acid levels and increases the risk of gout (Huang et al., 2021).

Although these observational studies and case reports have established a link between PPIs and elevated uric acid, they have limitations: small sample sizes (141 cases), reliance on medical insurance databases (lacking direct clinical test data and comprehensive multivariate analysis of laboratory indicators). These studies only preliminarily establish the PPI-hyperuricemia association without exploring quantitative relationships of risk factors or clinical prediction methods, resulting in a lack of effective clinical risk assessment tools. Nomogram prediction models, which integrate quantitative weights of multiple factors to generate individualized adverse event probabilities with convenience and high accuracy, have been widely applied in predicting disease-related adverse events (Mei et al., 2022; Ohori et al., 2009). This is a retrospective cohort study that collected medication history, baseline data, and laboratory indicators of inpatients. The aim was to verify the correlation between PPIs and hyperuricemia and screen independent risk factors for elevated uric acid after PPI use in patients with gastrointestinal bleeding via logistic regression and LASSO regression. A Nomogram model was constructed to provide quantitative risk assessment tools for clinical use, guide the rational use of PPIs, and facilitate early intervention of hyperuricemia.

## 2 Methods

### 2.1 Study design and patient collection

This cohort study was conducted at the Department of Pharmacy, West China Hospital, Sichuan University. We collected medical records of inpatients with gastrointestinal bleeding admitted from August 2022 to August 2025. According to the *Chinese multi-disciplinary consensus on the diagnosis and treatment of hyperuricemia and its related diseases*, hyperuricemia is defined as follows: under normal dietary conditions, hyperuricemia is diagnosed when fasting serum uric acid levels exceed 420 μmol/L (7 mg/dL) on two separate occasions (Multi-Disciplinary Expert Task Force on HIts Related, 2017). The patients included in this study were required to meet the diagnostic criteria for upper gastrointestinal and ulcer bleeding and acute lower gastrointestinal bleeding as set by the American College of Gastroenterology (Sengupta et al., 2023; Laine et al., 2021). Short-term inpatients (<7 days) were excluded, given their insufficient laboratory data and existing evidence that gout risk peaks within 30 days after PPIs treatment (Zhu et al., 2023). The exclusion criteria were as follows: hospital stay <7 days; hyperuricemia at baseline; PPIs use within 3 months before hospitalization; complicated with malignant tumors or undergoing dialysis; and incomplete laboratory test results. Baseline serum uric acid levels are defined as those below 420 μmol/L; baseline testing is performed upon admission, and the first abnormal result is used to determine the outcome.

### 2.2 Information collection and definitions

We extracted data from patients’ medical records, including demographics, diagnostic information, physical exams, laboratory test data, and medication records.

The PPIs we included are esomeprazole, ilaprazole, omeprazole, and pantoprazole. Other comorbidities included coronary artery disease, cerebrovascular accident, heart failure, chronic kidney disease, chronic liver disease, and rheumatoid arthritis.

Patients’ first hospitalization time was defined as the baseline, and discharge time as the endpoint. Patients who received PPI treatment for at least 7 days were categorized as PPI users, while those without PPI exposure were defined as PPI non-users. The outcome was the development of new-onset hyperuricemia. The continuous data were converted into binary variables based on the median, and the patients were divided into different subgroups. Patients were divided into subgroups based on binary variables derived from the median split of numerical test indicators. All patients were randomly divided into a training group and a validation group at a ratio of 7:3.

### 2.3 Statistical analysis

Data analysis and model development were performed using SPSS software (version 24.0, SPSS, IBM, United States) and R software (version 4.5.0, the R Core Team, United States) were used to conduct the data analysis and model development. The Shapiro-Wilk test was applied to test the normality of continuous variables. Non-normally distributed variables were described by the median and interquartile range (IQR) and compared using the Mann-Whitney U test. Categorical variables were described by their count (percentage) and compared using the χ^2^ test or Fisher’s exact test. A binary logistic regression model was used to calculate the odds ratio (OR) and the corresponding 95% confidence interval (CI). The Propensity Score Matching (PSM) model included the recipient’s gender, age, hospital time, serum creatinine, and uric acid at baseline. Using the PSM model, a caliper value of 0.02 was set, and 1:1 nearest-neighbor matching was performed. We then conducted a secondary analysis of the main results associated with the relationship between PPI and new-onset hyperuricemia patients. The LASSO regression method was employed as the feature selection method for the hyperuricemia prediction model. The regularization parameter λ was selected via 10-fold cross-validation. The value of λ was chosen such that the average cross-validation error fell within one standard error (1SE), thereby reducing the risk of model overfitting. Subsequently, a nomogram prediction model was constructed using the 10 non-zero variables identified by LASSO as predictors. To address potential class imbalance (i.e., where the sample size of the minority class is significantly smaller than that of the majority class), SMOTE sampling was applied only to the training set. K-fold cross-validation was employed for internal validation of the model. Discriminatory performance was assessed using the area under the receiver operating characteristic curve (AUC), with 1 − specificity on the x-axis (false positive rate) and sensitivity on the y-axis (true positive rate) (an AUC between 0.50 and 0.70 indicates low accuracy; 0.71 to 0.90 indicates moderate accuracy; above 0.90 indicates high accuracy). Calibration was assessed using the Hosmer-Lemeshow test (P > 0.05 indicates good calibration) and calibration curves. Furthermore, decision curve analysis (DCA) was employed to calculate the net benefit at different threshold probabilities, comparing the model against ‘all interventions’ and ‘no intervention’ strategies to evaluate the model’s clinical utility. A two-sided p-value of less than 0.05 is considered statistically significant.

## 3 Results

### 3.1 Baseline characteristics of the patients

**FIGURE 1:**
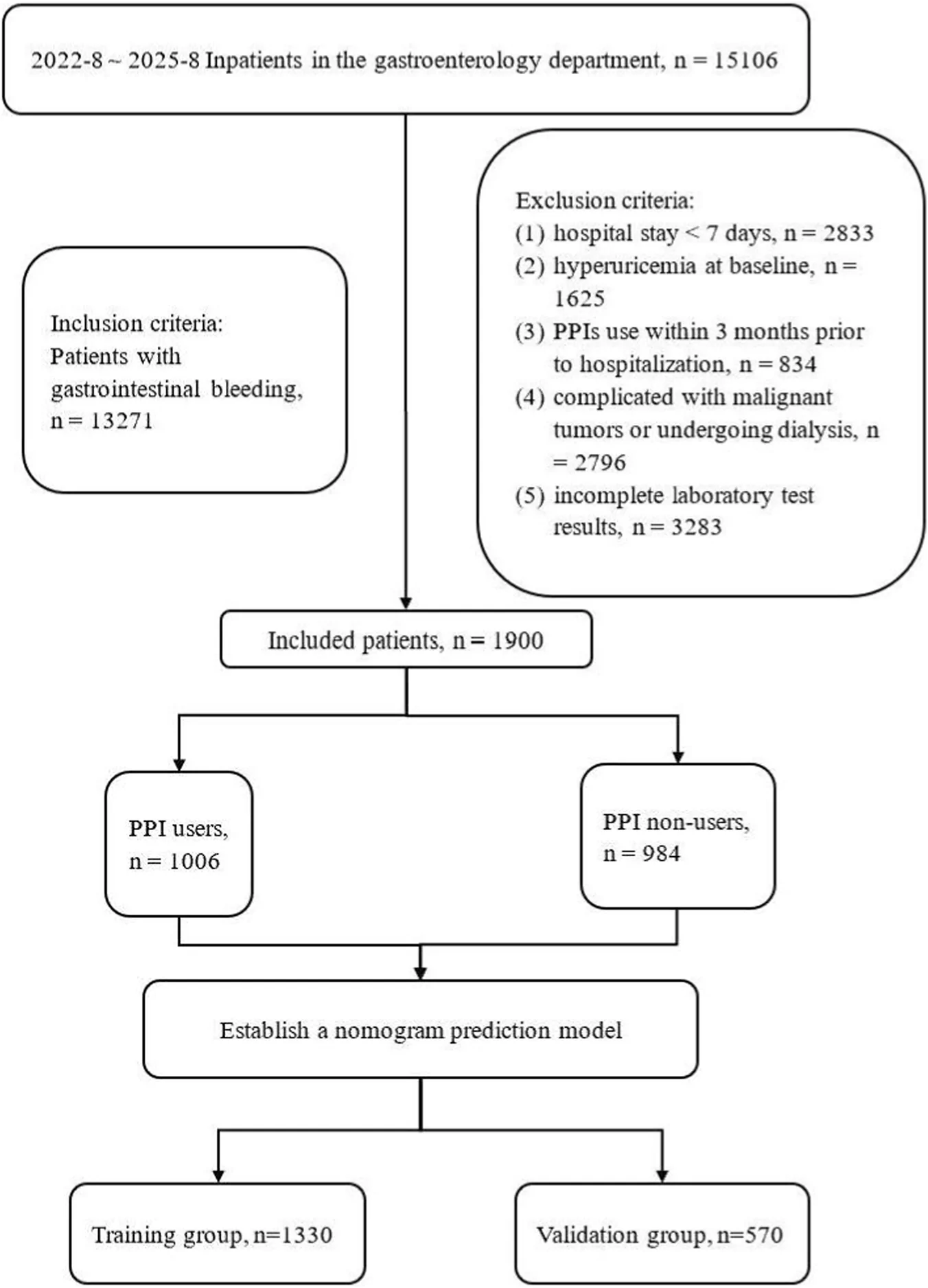
Flow chart of the study design.

From August 2022 to August 2025, a total of 13,271 patients with gastrointestinal bleeding were included. After excluding the cases that did not meet the criteria, a final total of 1,900 patients was included. Among 1,006 were PPI users, and 984 were PPI non-users. All patients were randomly divided into a training set (1,330 cases) and a validation set (570 cases) at a ratio of 7:3. A flowchart of patient enrollment is shown in Figure 1.

The baseline characteristics of the PPI-user and PPI non-user groups are listed in Table 1. Compared with non-users, PPI users had a higher proportion of males (*P* = 0.011) and longer hospital time (*P* < 0.001). Laboratory results showed that PPI users had lower serum calcium levels, higher serum creatinine levels, and lower total cholesterol levels than non-users. In addition, PPI users tend to use more diuretics, β-blockers, calcium channel blockers (CCBs) and non-steroidal anti-inflammatory drugs (NSAIDs). There were no discernible differences in baseline features between the training and validation sets, according to baseline consistency analysis (Supplementary Table 1). After propensity score matching, gender, age, serum creatinine, and uric acid at baseline were balanced between PPI users and non-users. A total of 1610 patients were matched, including 805 patients in the PPI use group and 805 patients in the non-use group (Supplementary Table 2). The results show that PPI users had longer hospital stays. The laboratory results show that the serum calcium levels and total cholesterol levels of PPI users are lower. Users of PPIs tend to use more diuretics, beta-blockers, CCBs, and NSAIDs.

**TABLE 1:** Baseline clinical characteristics of hospitalized patients.

Variable	Non-users (n = 984)	PPI users (n = 1006)	*P*
Gender, n (%)			
Famale	276 (30.9)	367 (36.5)	**0.011**
Male	618 (69.1)	639 (63.5)	
Age, years (IQR)	59.00 (47.00,71.00)	56.00 (43.00,68.00)	**0.005**
BMI (IQR)	23.14 (20.71,25.71)	23.04 (20.45,25.71)	0.531
Hospital time, day (IQR)	14.00 (10.00,22.00)	20.00 (12.00,33.00)	**< 0.001**
Alcohol, n (%)	127 (14.2)	158 (15.7)	0.396
Smoking, n (%)	185 (20.7)	222 (22.1)	0.501
Blood test indicators			
Uric acid, μmol/L (IQR)	261.00 (175.75,328.25)	245.50 (165.00,325.00)	0.079
Serum calcium, mmol/L (IQR)	2.09 (1.96,2.20)	2.06 (1.96,2.17)	**0.012**
Serum creatinine, μmol/L (IQR)	81.00 (65.00,107.00)	84.00 (66.00,125.00)	**0.015**
Globulin, g/L (IQR)	23.50 (19.78,27.10)	23.20 (19.20,27.40)	0.550
Triglyceride, mmol/L (IQR)	1.25 (0.87,1.91)	1.34 (0.93,1.92)	0.099
Total cholesterol, mmol/L (IQR)	3.23 (2.50,3.98)	3.08 (2.32,3.86)	**0.002**
Comorbidities, n (%)			
Hypertension	358 (37.8)	350 (34.8)	0.188
Diabetes mellitus	219 (24.5)	256 (25.4)	0.671
Hyperlipidemia	131 (14.7)	134 (13.3)	0.441
Other comorbidities	256 (28.6)	292 (29.0)	0.891
Medication, n (%)			
Diuretics	106 (11.9)	644 (64.0)	**< 0.001**
ACEI/ARBs	4 (0.4)	2 (0.2)	0.429
β-blockers	33 (3.7)	184 (18.3)	**< 0.001**
CCBs	64 (7.2)	248 (24.7)	**< 0.001**
NSAIDs	63 (7.0)	267 (26.5)	**< 0.001**
Types of PPIs, n (%)			
Esomeprazole	—	846 (84.1)	—
Ilaprazole	—	424 (42.1)	—
Omeprazole	—	30 (3.0)	—
Pantoprazole	—	67 (6.7)	—

Abbreviation: IQR, interquartile range; ACEI/ARB, Angiotensin-Converting Enzyme Inhibitor/Angiotensin II, receptor blocker; CCB, calcium channel blockers; NSAID, Non-Steroidal Anti-Inflammatory Drugs; Other comorbidities, Coronary artery disease, Cerebrovascular accident; Heart failure, Chronic kidney disease, Chronic liver disease, Rheumatoid arthritis; PPI, proton pump inhibitor.

Values in bold indicate a p-value < 0.05, indicating a statistically significant difference.

### 3.2 Association between PPIs and hyperuricemia

**FIGURE 2:**
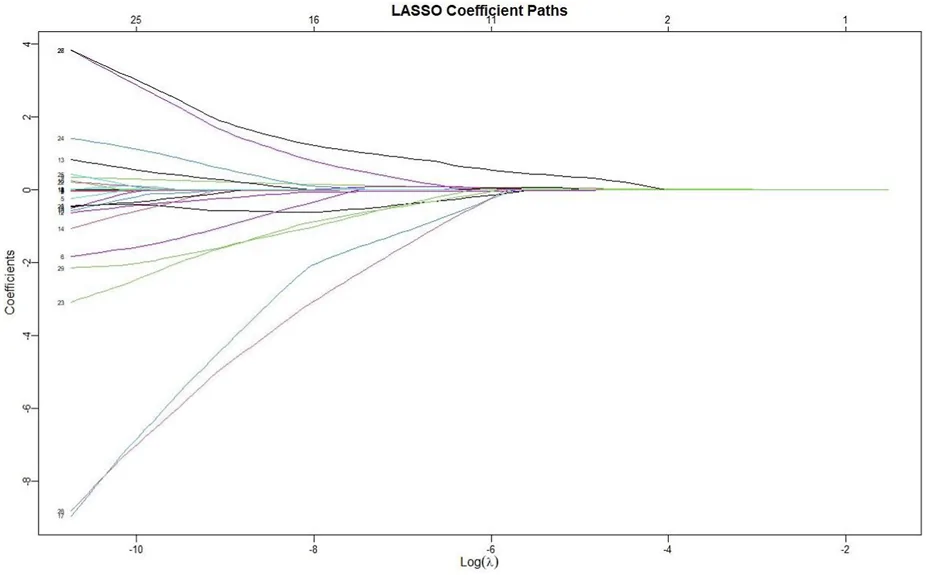
LASSO path.

**FIGURE 3:**
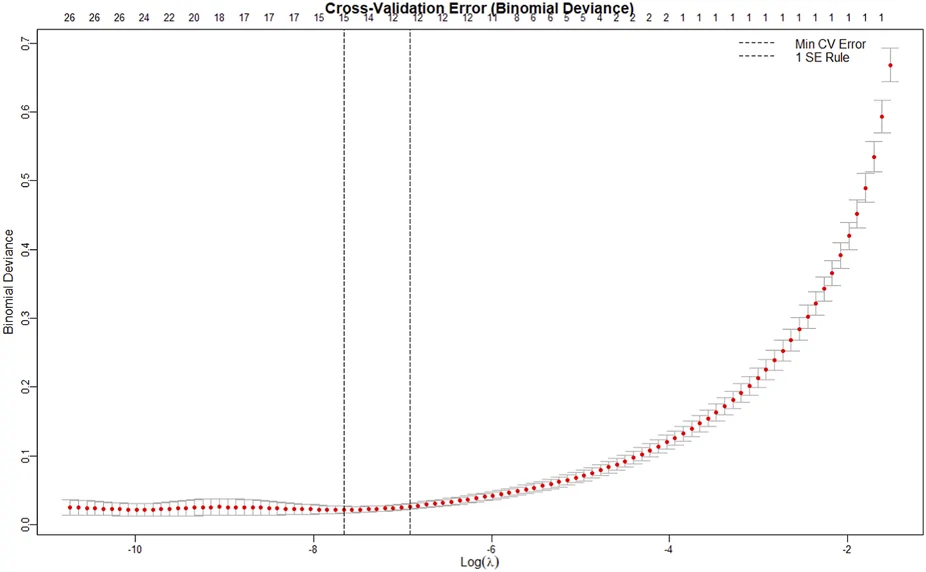
LASSO cross‐path.

A total of 199 patients (10.5%) developed new-onset hyperuricemia among all patients. Considering potential correlations between risk factors, LASSO regression was used to screen predictors of hyperuricemia. As the penalty coefficient λ changes, all variables in the model are gradually compressed (Figure 2). To select the optimal variables and avoid overfitting, the ten-fold cross-validation method was used to select the smallest value 1SE (λ = 0.00099) as the optimal penalty coefficient, and finally, 10 non-zero coefficient predictors were selected (Figure 3).

We conducted a stratified analysis of 18 factors such as gender, age, hospital time, BMI, smoking, alcohol, serum creatinine, uric acid, triglyceride, ACEI/ARB, β-blocker, NSAID, CCB, diuretic, hypertension, diabetes, hyperlipidemia, and other comorbidities (Table 2). In most subgroups, the incidence of new-onset hyperuricemia in the PPI group was higher than that in the non-PPI group. However, there was no significant difference in the younger patients (Age ≤30 years). Univariate and multivariate logistic regression analyses were performed on 19 influencing factors, including those identified by LASSO analysis (Table 3). The multivariate regression analysis revealed that PPI exposure was associated with the development of new-onset hyperuricemia, with an adjusted OR (95% CI) of 4.86 (3.04, 7.78). The relationship between PPI and hyperuricemia was also explored in patients who were further matched in PSM (Table 4). The use of PPI was associated with hyperuricemia, with adjusted OR (95% CI) of 4.35 (2.67, 7.07).

**TABLE 2:** The relationship between PPI exposure and hyperuricemia in subgroups of patients.

Variable	Non-users	PPI users	Or (95% CI)	Adjusted OR (95% CI)
	Normal/Hyperuricemia	Normal/Hyperuricemia		
Gender				
Male	264/12	304/63	4.56 (2.41,8.64)	2.87 (1.30,6.33)
Female	598/20	535/104	5.81 (3.55,9.51)	6.25 (3.48,11.25)
Age, years				
≤30	94/4	106/3	0.67 (0.15,3.05)	1.28 (0.12,13.41)
>30	768/28	733/164	6.14 (4.06,9.28)	5.02 (3.08,8.18)
Hospital time, day				
≤17	530/15	383/55	5.07 (2.82,9.12)	5.10 (2.56,10.14)
>17	332/17	456/112	4.80 (2.83,8.14)	4.30 (2.22,8.33)
BMI				
≤23.12	426/16	416/89	5.70 (3.29,9.86)	5.02 (2.64,9.54)
>23.12	436/16	423/78	5.03 (2.89,8.75)	4.44 (2.24,8.79)
Smoking				
No	687/22	645/139	6.73 (4.24,10.69)	7.44 (4.27,12.98)
Yes	175/10	194/28	2.53 (1.19,5.35)	2.79 (1.32,6.11)
Alcohol				
No	743/24	699/149	6.60 (4.24,10.28)	6.62 (3.90,11.23)
Yes	119/8	140/18	1.91 (0.80,4.56)	1.01 (1.00,5.89)
Serum creatinine, μmol/L				
≤82	459/10	453/41	4.15 (2.06,8.39)	3.48 (1.53,7.93)
>82	403/22	386/126	5.49 (3.11,9.69)	5.98 (3.72,9.61)
Uric acid, μmol/L				
≤252	450/24	364/113	5.82 (3.67,9.24)	4.41 (2.55,7.62)
>252	412/8	475/54	5.86 (2.75,12.45)	5.81 (2.40,14.09)
Triglyceride, mmol/L				
≤1.3	458/17	393/86	5.90 (3.45,10.09)	4.54 (2.39,8.62)
>1.3	404/15	446/81	4.89 (2.77,8.63)	4.95 (2.49,9.83)
ACEI/ARB				
No	858/32	838/166	5.31 (3.6,7.85)	4.60 (2.89,7.34)
Yes	4/0	1/1	—	—
β-blocker				
No	829/32	694/128	4.78 (3.2,7.13)	4.59 (2.81,7.51)
Yes	33/0	145/39	—	—
NSAID				
No	803/28	633/106	5.26 (3.12,8.87)	4.80 (3.13,7.38)
Yes	59/4	206/61	4.37 (1.53,12.51)	3.04 (1.00,9.22)
CCB				
No	801/29	649/109	4.64 (3.04,7.08)	4.49 (2.67,7.54)
Yes	61/3	190/58	6.21 (1.88,20.52)	4.09 (1.13,14.82)
Diuretic				
No	761/27	319/43	3.80 (2.31,6.26)	4.90 (2.75,8.72)
Yes	101/5	520/124	4.82 (1.92,12.08)	4.61 (1.78,11.96)
Hypertension				
No	536/20	577/79	5.50 (3.55,8.53)	4.31 (2.56,7.27)
Yes	326/12	262/88	5.11 (2.16,12.11)	6.95 (2.31,12.87)
Diabetes mellitus				
No	649/26	637/113	4.43 (2.85,6.88)	3.87 (2.30,6.52)
Yes	213/6	202/54	9.49 (4.00,12.54)	8.29 (2.81,14.47)
Hyperlipidemia				
No	738/25	735/137	4.12 (2.50,6.79)	2.91 (1.61,5.24)
Yes	124/7	104/30	3.67 (2.22,6.08)	2.90 (1.61,5.25)
Other comorbidities				
No	618/20	630/84	9.13 (4.89,17.04)	9.31 (4.29,20.20)
Yes	244/12	209/83	8.08 (4.29,15.21)	10.31 (4.70,22.66)

The confounding factors included in the multivariate regression analysis were gender (female = 1, male = 0), smoking (yes = 1, no = 0), alcohol (yes = 1, no = 0), ACEI/ARBs (yes = 1, no = 0), β-blockers (yes = 1, no = 0), NSAIDs (yes = 1, no = 0), CCBs (yes = 1, no = 0), diuretics (yes = 1, no = 0), Hypertension (yes = 1, no = 0), diabetes mellitus (yes = 1, no = 0) hyperlipidemia (yes = 1, no = 0), other comorbidities (yes = 1, no = 0).

**TABLE 3:** Logistic regression analysis of hyperuricemia in all patients.

Variables	Univariate		Multivariate	
	Odds ratio (95% CI)	*P*	Odds ratio (95% CI)	*P*
Gender	0.83 (0.61,1.12)	0.226	0.91 (0.65,1.27)	0.584
Age	1.02 (1.01,1.03)	**< 0.001**	1.01 (1.00,1.03)	**0.006**
Hospital time	1.01 (1.01,1.02)	**< 0.001**	1.01 (1.00,1.02)	**0.004**
BMI	1.01 (0.97,1.04)	0.673	0.98 (0.94,1.02)	0.322
Smoking	0.85 (0.59,1.24)	0.398	0.87 (0.53,1.42)	0.573
Alcohol	0.84 (0.54,1.29)	0.420	0.90 (0.51,1.61)	0.733
Serum creatinine	1.00 (1.00,1.00)	**< 0.001**	1.00 (1.00,1.00)	**< 0.001**
Uric acid	1.01 (1.01,1.01)	**< 0.001**	1.01 (1.00,1.01)	**< 0.001**
Triglyceride	0.98 (0.89,1.08)	0.659	0.91 (0.77,1.07)	0.258
PPIs	5.36 (3.63,7.92)	**< 0.001**	4.86 (3.04,7.78)	**< 0.001**
ACEI/ARBs	1.71 (0.2,14.74)	0.624	0.94 (0.08,10.57)	0.959
β-blockers	2.09 (1.42,3.06)	**< 0.001**	0.79 (0.50,1.23)	0.296
NSAIDs	2.63 (1.90,3.63)	**< 0.001**	1.38 (0.94,2.02)	0.099
CCBs	2.55 (1.84,3.55)	**< 0.001**	0.97 (0.63,1.49)	0.894
Diuretics	3.21 (2.36,4.36)	**< 0.001**	1.29 (0.87,1.92)	0.209
Hypertension	1.91 (1.42,2.57)	**< 0.001**	1.25 (0.85,1.84)	0.253
Diabetes mellitus	1.34 (0.97,1.85)	0.077	0.98 (0.67,1.42)	0.902
Hyperlipidemia	1.48 (1.01,2.17)	**0.047**	1.69 (1.05,2.72)	**0.029**
Other comorbidities	2.52 (1.87,3.39)	**< 0.001**	1.46 (1.02,2.10)	**0.039**

Abbreviation: CI, confidence interval; PPI, proton pump inhibitor; CCB, Calcium Channel Blockers. The confounding factors included in the multivariate regression analysis were gender (female = 1, male = 0), age (continuous), hospital time (continuous), BMI (continuous), smoking (yes = 1, no = 0), alcohol (yes = 1, no = 0), serum creatinine (continuous), uric acid (continuous), triglyceride (continuous), PPI (yes = 1, no = 0), ACEI/ARBs (yes = 1, no = 0), β-blockers (yes = 1, no = 0), NSAIDs (yes = 1, no = 0), CCBs (yes = 1, no = 0), diuretics (yes = 1, no = 0), Hypertension (yes = 1, no = 0), diabetes mellitus (yes = 1, no = 0) hyperlipidemia (yes = 1, no = 0), other comorbidities (yes = 1, no = 0).

Values in bold indicate a p‐value < 0.05, indicating a statistically significant difference.

**TABLE 4:** Logistic regression analysis of hyperuricemia in PSM patients.

Variables	Univariate		Multivariate	
	Odds ratio (95% CI)	*P*	Odds ratio (95% CI)	*P*
Gender	0.86 (0.61,1.21)	0.387	0.89 (0.61,1.28)	0.522
Age	1.02 (1.01,1.03)	**< 0.001**	1.01 (1.00,1.03)	0.016
Hospital time	1.01 (1.00,1.02)	0.002	1.02 (1.01,1.02)	0.001
BMI	1.02 (0.98,1.06)	0.431	0.98 (0.94,1.03)	0.413
Smoking	0.91 (0.60,1.36)	0.639	0.94 (0.55,1.61)	0.816
Alcohol	0.90 (0.57,1.43)	0.654	0.95 (0.51,1.77)	0.877
Serum creatinine	1.00 (1.00,1.00)	**< 0.001**	1.00 (1.00,1.00)	0.002
Uric acid	1.01 (1.00,1.01)	**< 0.001**	1.01 (1.00,1.01)	**< 0.001**
Triglyceride	0.97 (0.86,1.11)	0.677	0.90 (0.74,1.09)	0.277
PPIs	4.65 (3.12,6.94)	**< 0.001**	4.35 (2.67,7.07)	**< 0.001**
ACEI/ARBs	—	—	—	—
β-blockers	2.07 (1.32,3.22)	0.001	0.86 (0.52,1.43)	0.553
NSAIDs	2.35 (1.62,3.41)	**< 0.001**	1.33 (0.87,2.04)	0.195
CCBs	2.29 (1.57,3.33)	**< 0.001**	0.95 (0.59,1.53)	0.831
Diuretics	3.15 (2.25,4.41)	**< 0.001**	1.36 (0.88,2.10)	0.162
Hypertension	1.75 (1.26,2.43)	0.001	1.07 (0.70,1.64)	0.745
Diabetes mellitus	1.45 (1.02,2.06)	0.040	1.14 (0.76,1.70)	0.540
Hyperlipidemia	1.35 (0.87,2.09)	0.175	1.39 (0.83,2.33)	0.217
Other comorbidities	2.53 (1.82,3.51)	**< 0.001**	1.57 (1.06,2.32)	0.026

Abbreviation: The Propensity Score Matching (PSM) model included recipient’s gender, age, hospital time, serum creatinine and uric acid at baseline. CI, confidence interval; PPI, proton pump inhibitor; CCB, Calcium Channel Blockers. The confounding factors included in the multivariate regression analysis were gender (female = 1, male = 0), age (continuous), hospital time (continuous), BMI (continuous), smoking (yes = 1, no = 0), alcohol (yes = 1, no = 0), serum creatinine (continuous), uric acid (continuous), triglyceride (continuous), PPI (yes = 1, no = 0), ACEI/ARBs (yes = 1, no = 0), β-blockers (yes = 1, no = 0), NSAIDs (yes = 1, no = 0), CCBs (yes = 1, no = 0), diuretics (yes = 1, no = 0), Hypertension (yes = 1, no = 0), diabetes mellitus (yes = 1, no = 0) hyperlipidemia (yes = 1, no = 0), other comorbidities (yes = 1, no = 0).

Values in bold indicate a p‐value < 0.05, indicating a statistically significant difference.

### 3.3 Establishment and validation of a high uric acid prediction model

**FIGURE 4:**
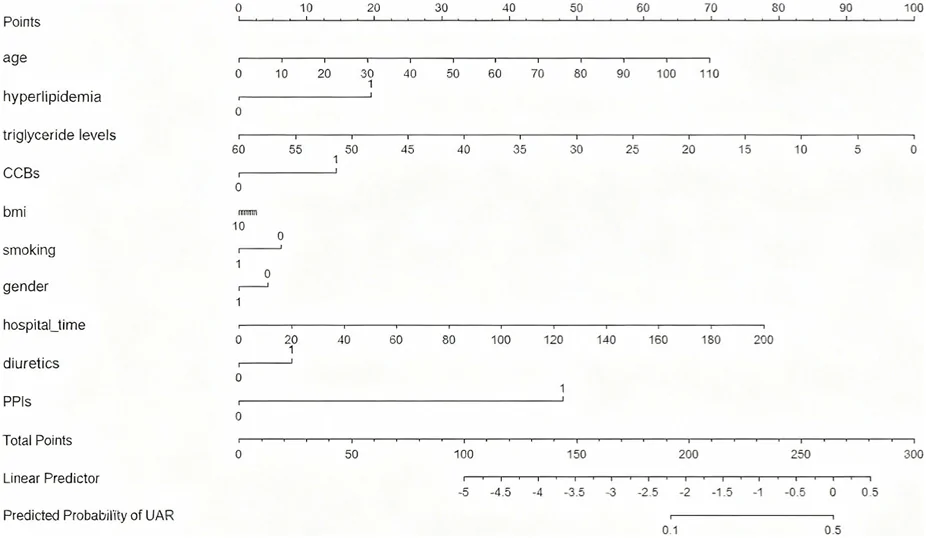
Nomogram for predicting the risk of hyperuricemia caused by PPIs.

To intuitively quantify the risk of hyperuricemia, this study incorporated 10 screened predictors into the construction of a nomogram model. Each predictive variable was assigned an independent scoring line to weigh in the risk prediction model. For clinical application, individual variable scores are derived by aligning patient data with the corresponding axis, and the total score is then converted into a predicted probability via the nomogram’s risk scale. The total score is positively correlated with the individual’s risk of hyperuricemia (Figure 4).

#### 3.3.1 Validation of model discrimination

**FIGURE 5:**
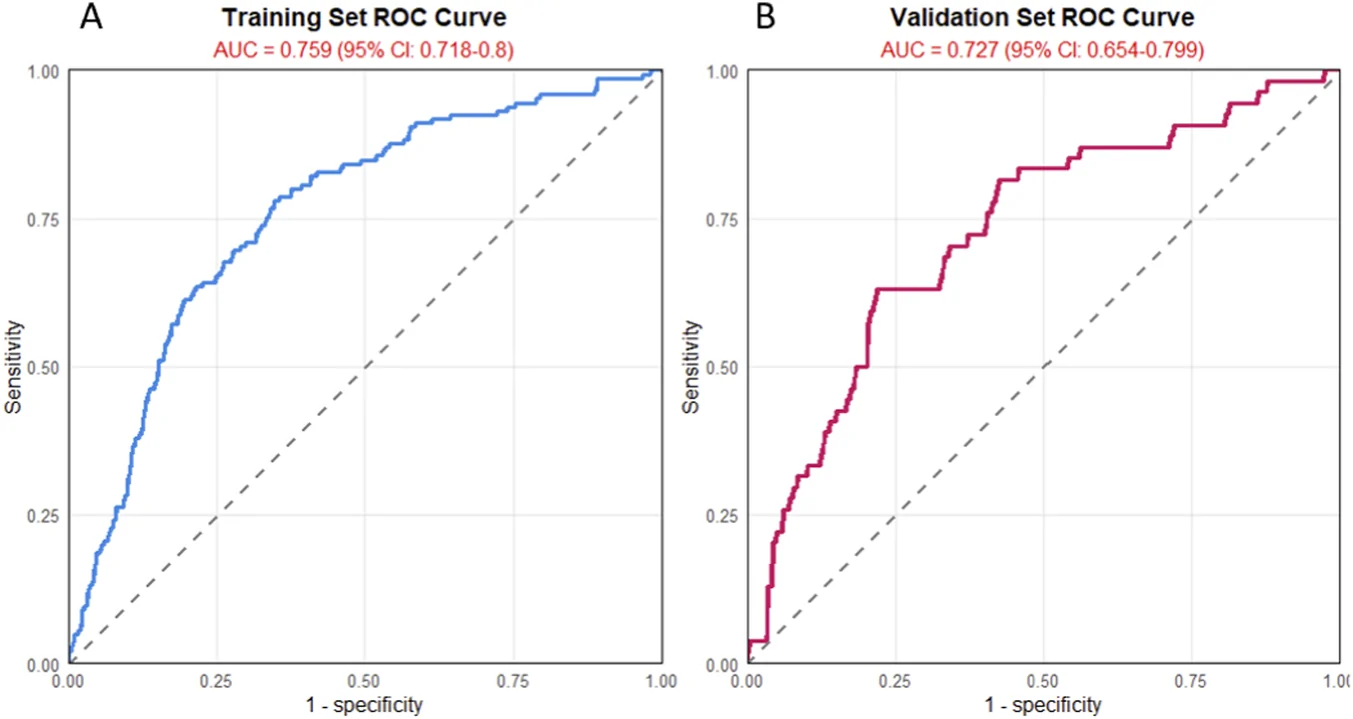
The ROC curves. **(A)** Training set ROC curve, **(B)** Validation set ROC.

Subject AUC was used to evaluate the discriminative ability of the model, and ROC curves were drawn with specificity as the horizontal axis and sensitivity as the vertical axis. The results showed that the AUC of the training set was 0.759 (95% CI: 0.718, 0.800), and the AUC of the validation set was 0.727 (95% CI: 0.654, 0.799), indicating that the model has good discriminative efficacy for the incidence risk of hyperuricemia (Figure 5).

#### 3.3.2 Validation of model calibration

**FIGURE 6:**
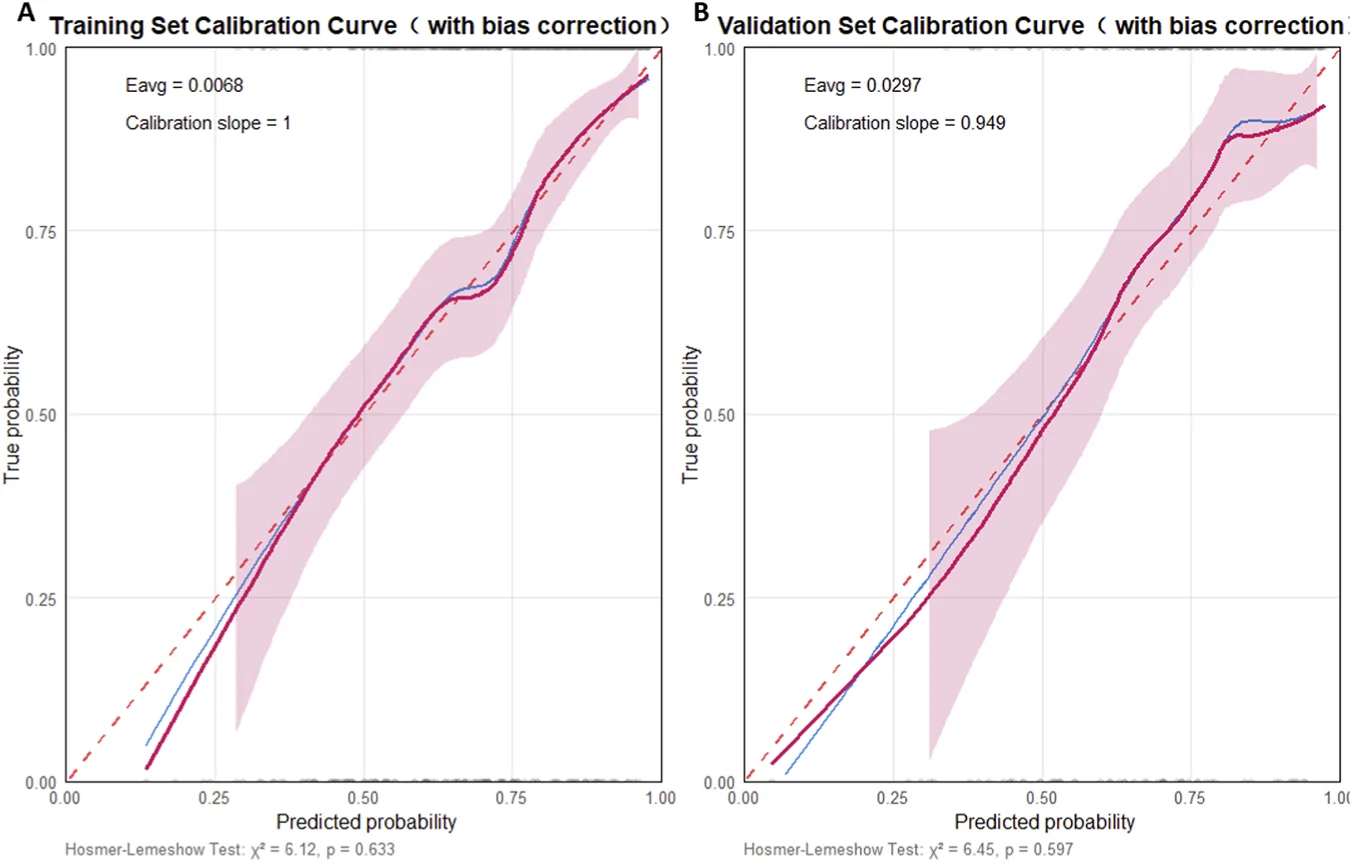
The Calibration curves. **(A)** Training set Calibration curve, **(B)** Validation set Calibration curve.

The Hosmer-Lemeshow test was adopted for assessing model goodness-of-fit and calibration. The test results showed that the *P*-value of the training set was 0.633 and that of the validation set was 0.597 (*P* > 0.05), indicating good model fit. The results showed that the calibration curves of both the training set and the validation set were close to the ideal line, with slopes of 1.000 and 0.949, respectively, demonstrating that the model has good calibration ability (Figure 6).

#### 3.3.3 Validation of model efficacy

**FIGURE 7:**
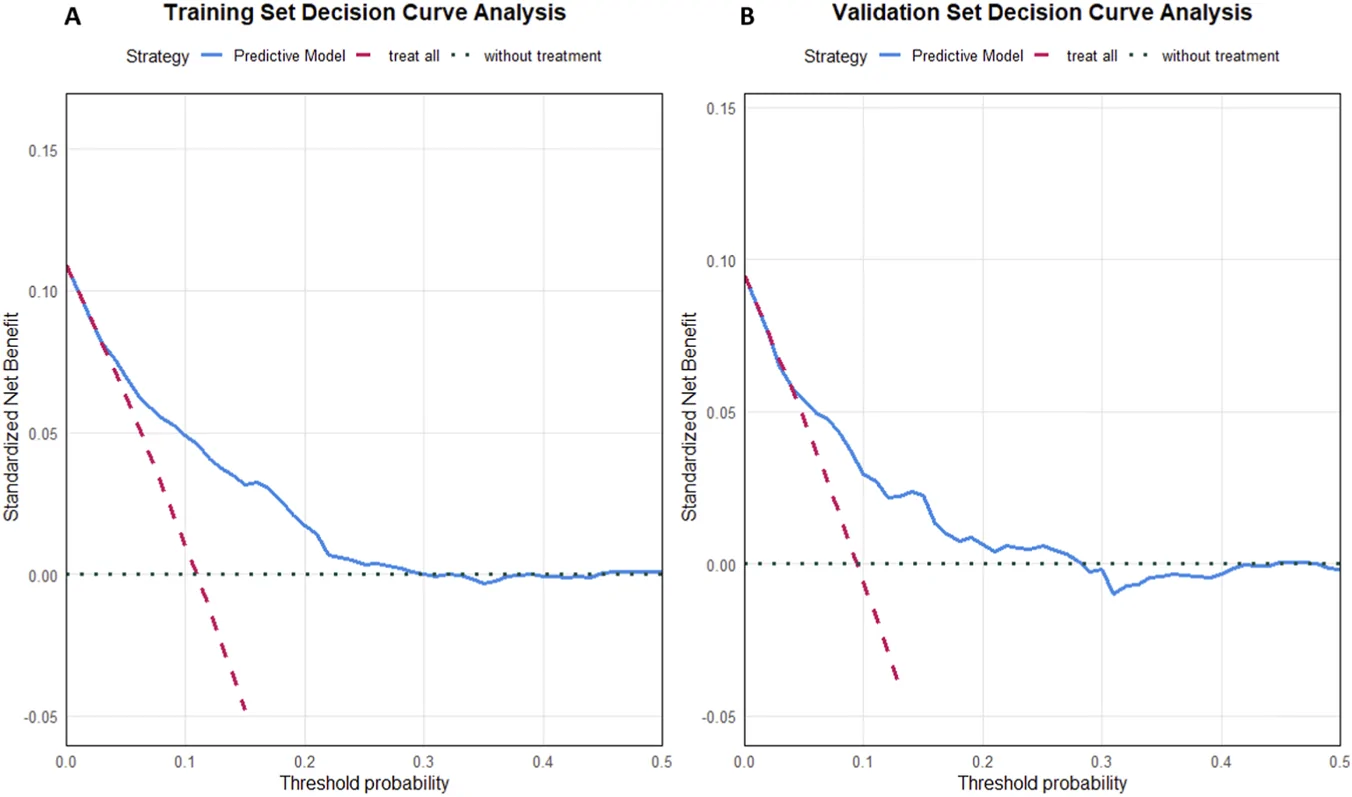
The decision curves analysis. **(A)** Training set decision curve analysis, **(B)** Validation set decision curve analysis.

DCA was utilized to evaluate the clinical net benefit of the model (Figure 7). The results show that the model’s net benefit exceeded both the net benefit of no treatment and the net benefit of treatment in both the training and validation sets. The prediction model has good clinical application value.

## 4 Discussion

This study explored the association between PPI exposure and serum uric acid levels and identified a positive correlation between PPI use and incident hyperuricemia among patients hospitalized for gastrointestinal bleeding. Early case reports documenting episodes of acute gout concurrent with PPI administration were initially published in *The Lancet*, and subsequent clinical analyses have further investigated this correlational relationship (Kraus and Flores-Suarez, 1995). A study utilizing the Taiwan insurance database showed that the risk of gout varies among different PPI subtypes. The esomeprazole users had a 30% increased risk (95% CI: 1.0, 1.6), and the risk peaked within 30 days after treatment (95% CI: 1.4, 1.9) (Zhu et al., 2023). A retrospective study found that for every 10-unit increase in PPI dosage, the risk of acute gout attacks increased by 3% (95% CI: 1.01, 1.04) (Xu, 2024). However, existing research findings remain inconsistent. A UK-based nested case-control study enrolling 53,000 participants detected no statistically significant association between omeprazole use and elevated gout incidence. Even so, the authors hypothesized that omeprazole might occasionally coincide with gout onset in specific subpopulations, while the biological pathways underlying this observed correlation remain poorly elucidated (Meier and Jick, 1997). Multiple hypothetical biological pathways have been proposed to rationalize the observed positive association between PPI exposure and elevated serum uric acid concentrations. First, PPI-mediated suppression of gastric and renal H^+^/K^+^-ATPase activity is postulated to accelerate the breakdown of surplus ATP into adenine, a precursor substrate for uric acid biosynthesis (Schiffl et al., 2020). Moreover, inhibition of renal H^+^/K^+^-ATPase is theorized to lower intratubular pH and diminish uric acid solubility, which may in turn attenuate renal uric acid excretion (Yuan et al., 2018; Callaghan et al., 1995; Celik et al., 2017). Emerging evidence suggests PPI exposure may correlate with aggravated insulin resistance via alterations in hypomagnesemia and insulin-like growth factor-1 (IGF-1) homeostasis; insulin resistance itself is known to facilitate renal tubular uric acid reabsorption, which could partially explain the observed correlation (Barchetta et al., 2015; Lima Mde et al., 2009; Tanaka et al., 2022).

Subgroup analyses revealed that the effect of PPI on uric acid levels was not significant in younger patients (age ≤ 30 years). This may be explained by the mechanism through which PPI-induced insulin resistance promotes renal urate reabsorption (Lima Mde et al., 2009). Young individuals generally have a lower prevalence of insulin resistance, allowing them to buffer the metabolic impact of PPI through stronger homeostatic capacity. Age itself is a known determinant of uric acid levels. In a cohort study conducted in 2014, it was shown that hyperuricemia prevalence rates of 18.6% (18–39 years), 20.2% (40–59 years), 27.7% (60–80 years), and 43.0% (>80 years), confirming an age-dependent increase (Kumar et al., 2018). Additionally, the lower rates of concomitant medication use and comorbidities in younger patients compared with middle-aged and elderly individuals may further account for the lack of significant PPI-associated uric acid elevation in this group. In addition to PPIs, other factors affecting uric acid are worth exploring. The study identified 10 independent risk factors for hyperuricemia, including gender, age, hospital time, BMI, smoking, triglyceride, PPI, CCBs, diuretics, and hyperlipidemia. Song et al.'s research found that age, BMI, smoking, coastal residence, ethnicity, diabetes, hypertension, and total cholesterol levels were independently associated with hyperuricemia (Song et al., 2022). Furthermore, genome-wide association studies (GWAS) have confirmed that more than 200 genetic loci are associated with hyperuricemia, and such associations exhibit heterogeneity across different genders and ethnicities (Boocock et al., 2020; Jansen et al., 2019). Diuretics can elevate serum uric acid by reducing renal urate excretion and interfering with urate transporters, thereby potentially inducing gout (Dalbeth et al., 2019). Additionally, medications such as β-blockers, angiotensin-converting enzyme inhibitors, immunosuppressants, and low-dose aspirin may increase gout risk (Choi et al., 2012; Asghari et al., 2024). In the pilot phase of this study, some of these drugs were included. However, logistic regression did not reveal statistical significance. We hypothesize they may have latent hyperuricemic potential not captured by the model. In contrast, LASSO regression incorporates an L1 regularization term (penalty term) to effectively perform variable selection, mitigate multicollinearity, and reduce the risk of overfitting (Harris, 2021; Bayman and Dexter, 2021; Varady et al., 2023; Hepp et al., 2019; Xi et al., 2023). Thus, LASSO regression was used for predictor selection in this study.

The hyperuricemia prediction model established in this study was comprehensively evaluated from multiple dimensions. The constructed nomogram improved the model’s intuitiveness, thereby enabling clinicians to rapidly calculate the risk of hyperuricemia. This was achieved by assigning scores to each variable based on its value and summing up the total score according to the individual patient’s conditions (Balachandran et al., 2015). To address the data imbalance caused by the low proportion of positive cases in the sample, the synthetic minority oversampling technique (SMOTE) was adopted for correction (Ahmad et al., 2024). The calibration assessment showed that the Hosmer-Lemeshow test verified good model fit (training set *P* = 0.633, validation set *P* = 0.597). The calibration curve was highly consistent with the ideal reference line, indicating a strong agreement between the predicted and actual disease risks, which could prevent overestimation or underestimation of hyperuricemia occurrence (Fenlon et al., 2018). The DCA demonstrates that the corresponding blue curve of the model is higher than the two extreme curves of “total treatment” and “no treatment at all”. This finding confirms that the model has good clinical net benefits and practical clinical application value (Vick et al., 2021).

Uric acid is not only a key diagnostic marker for gout but has also been shown to be a significant risk factor and biomarker closely associated with the onset and progression of various diseases, including hyperglycemia, hyperlipidemia, hypertension, metabolic syndrome, cardiovascular and cerebrovascular diseases, and cancer (Kuwabara et al., 2023; Kuwabara et al., 2025; Dovell and Boffetta, 2018). In the field of metabolic diseases, hyperuricemia contributes to the onset and progression of type 2 diabetes, dyslipidemia, and metabolic syndrome through mechanisms such as inducing insulin resistance, inhibiting nitric oxide synthesis, and promoting the release of inflammatory factors from adipose tissue; it serves as an early warning sign of metabolic disorders (Vareldzis et al., 2024). Concerning cardiovascular disease, extensive epidemiological evidence indicates that hyperuricemia is independently associated with the risk of developing hypertension, coronary heart disease, heart failure, and stroke, as well as with poor prognosis. The underlying mechanisms involve multiple pathways, including oxidative stress, endothelial dysfunction, vascular remodeling, and the activation of inflammatory signaling pathways via the renin-angiotensin system (Kimura et al., 2021). Furthermore, a growing body of research indicates that abnormal uric acid levels are also associated with the development, progression, and prognosis of various cancers; the mechanisms underlying this may be related to chronic inflammation, oxidative stress imbalance, and abnormal regulation of cell proliferation (Li et al., 2026). Consequently, serum uric acid is no longer confined to the diagnosis of gout but serves as a comprehensive indicator reflecting the body’s metabolic homeostasis, inflammatory burden, and risk of multisystem diseases, holding significant clinical value for the early identification, risk assessment, and intervention management of various chronic conditions.

This study has several limitations. First, this was a retrospective study. Despite the large sample size, selection bias was present in the enrolled cohort. Since all included patients were hospitalized cases and those with short-term hospitalization (<7 days) were excluded, the enrolled population might be composed of patients with better compliance or more severe conditions, which fails to reflect the real efficacy of PPI in the overall population of patients with gastrointestinal bleeding. Second, although most information was collected from hospital medical records, potential confounding factors (diet, genetic factors, economic status, *etc.*) could not be fully controlled. Third, detailed data regarding in-hospital hemorrhage episodes, hemodynamic parameters, and nutritional status during hospitalization were not captured in this study. Although baseline confounders including admission diagnosis and BMI were standardized at enrollment, this study only assessed the crude correlation between PPI exposure and uric acid levels without adjusting for these confounders. Future investigations should adequately account for confounding biases introduced by post-admission therapeutic interventions and dynamic disease progression. Finally, this study lacks multicenter external validation, which may lead to insufficient generalizability and predictive performance of the established model. Nevertheless, this cohort study still has several strengths. To begin with, it was a large-scale retrospective study involving 1,900 Chinese patients with gastrointestinal bleeding. Logistic regression and LASSO regression were applied to explore the effect of PPI on serum uric acid levels. More importantly, this is the first study to establish a PPI exposure and new-onset hyperuricemia risk prediction indicator in patients with gastrointestinal bleeding. This tool will enable clinicians to calculate the total score and assess the risk of hyperuricemia before intervention, and further implement preventive measures to reduce the incidence of hyperuricemia.

## 5 Conclusion

This cohort study suggests a potential association between PPI use and new-onset hyperuricemia in hospitalized patients with gastrointestinal bleeding. To assess the risk of PPI-induced hyperuricemia, various factors related to hyperuricemia were incorporated, and a nomogram prediction model based on 10 predictors was developed and validated. The model demonstrated favorable predictive performance and clinical utility in comprehensive evaluations. The application of this prediction model may facilitate closer monitoring and early intervention, thereby assisting clinicians in the prevention of hyperuricemia among high-risk patients.

## Data Availability

The raw data supporting the conclusions of this article will be made available by the authors, without undue reservation.
