# Supplementary material for: Association between exposure to proton pump inhibitors and hyperuricemia: risk prediction model establishment and nomogram construction
Source: Front Pharmacol. 2026 Aug 7;17:1842574. doi: 10.3389/fphar.2026.1842574 (PMC13493512; doi:10.3389/fphar.2026.1842574)
Supplement: Supplementary file 1 [file Table1.docx]

Supplementary Material

# 1 Supplementary Tables 1

**Supplementary Tables 1** Comparison of Baseline Data Between Training and Validation Sets

| Variable | Training group  (n = 1330) | Validation group  (n = 570) | *P* |
| --- | --- | --- | --- |
| Gender, n (%) |  |  |  |
| Famale | 440(33.1) | 203(35.6) | 0.310 |
| Male | 890(66.9) | 367(65.0) |  |
| Age, years (IQR) | 58.00(45.00,70.00) | 57.00(44.00-69.00) | 0.620 |
| BMI (IQR) | 23.03(20.32,25.68) | 23.44(20.76,25.75) | **0.029** |
| Hospital time, day (IQR) | 17.00(11.00,28.00) | 17.00(11.00,28.00) | 0.780 |
| Alcohol, n (%) | 204(15.3) | 81(14.2) | 0.575 |
| Smoking, n (%) | 294(22.1) | 113(19.8) | 0.294 |
| Blood test indicators |  |  |  |
| Uric acid, μmol/L (IQR) | 251.00(174.00,326.00) | 254.00(163.25,332.00) | 0.745 |
| Serum calcium, mmol/L (IQR) | 2.07(1.98,2.21) | 2.08(1.99,2.23) | 0.563 |
| Serum creatinine, μmol/L (IQR) | 82.00(65.00,117.00) | 81.00(66.0,113.00) | 0.895 |
| Globulin, g/L (IQR) | 23.30(19.40,27.20) | 23.30(19.52,27.10) | 0.754 |
| Triglyceride, mmol/L (IQR) | 1.28(0.89,1.87) | 1.32(0.91,1.99) | 0.238 |
| Total Cholesterol, mmol/L (IQR) | 3.13(2.39,3.90) | 3.13(2.41,3.96) | 0.623 |
| Comorbidities, n (%) |  |  |  |
| Hypertension | 145(10.9) | 54(9.5) | 0.395 |
| Diabetes mellitus | 324(24.4) | 151(26.5) | 0.355 |
| Hyperlipidemia | 187(14.1) | 78(13.7) | 0.885 |
| Other comorbidities | 381(28.6) | 167(29.3) | 0.816 |
| Medication, n (%) |  |  |  |
| PPI | 719(54.1) | 287(50.4) | 0.151 |
| Diuretics | 532(40.0) | 218(38.2) | 0.506 |
| ACEI/ARB | 3(0.2) | 3(0.5) | 0.373 |
| β-blockers | 145(10.9) | 72(12.6) | 0.314 |
| CCBs | 220(16.5) | 92(16.1) | 0.882 |
| NSAIDs | 242(18.2) | 88(15.4) | 0.165 |
| Types of PPIs, n (%) |  |  |  |
| Esomeprazole | — | 846(84.1) | — |
| Ilaprazole | — | 424(42.1) | — |
| Omeprazole | — | 30(3.0) | — |
| Pantoprazole | — | 67(6.7) | — |

**Abbreviation:** IQR, interquartile range; ACEI/ARB, Angiotensin-Converting Enzyme Inhibitor / Angiotensin Ⅱ Receptor Blocker; CCB, Calcium Channel Blockers; NSAID, Non-Steroidal Anti-Inflammatory Drugs; Other comorbidities, Coronary artery disease, Cerebrovascular accident; Heart failure, Chronic kidney disease, Chronic liver disease, Rheumatoid arthritis; PPI, Proton pump inhibitor.

# 2 Supplementary Tables 2

**Supplementary Tables 2** Baseline clinical characteristics in PSM patients

| Variable | Non-PPI (n = 805) | PPI (n = 805) | *P* |
| --- | --- | --- | --- |
| Gender, n (%) |  |  |  |
| Famale | 260(32.3) | 258(32.0) | 0.957 |
| Male | 545(67.7) | 547(68.0) |  |
| Age, years (IQR) | 59.00(45,00,71.00) | 57.00(45.00,69.00) | 0.255 |
| BMI (IQR) | 23.03(20.52,25.66) | 23.03(20.45,25.71) | 0.763 |
| Hospital time, day (IQR) | 15.00(10.00,23.00) | 16.00(11.00,26.00) | **0.004** |
| Alcohol, n (%) | 117(14.5) | 131(16.3) | 0.369 |
| Smoking, n (%) | 164(20.4) | 177(22.0) | 0.464 |
| Blood test indicators |  |  |  |
| Uric acid, μmol/L (IQR) | 258.00(172.00,328.00) | 249.00(173.00,327.00) | 0.646 |
| Serum calcium, mmol/L (IQR) | 2.09(1.97,2.21) | 2.06(1.95,2.17) | **0.011** |
| Serum creatinine, μmol/L (IQR) | 81.00(65.00,108.00) | 83.00(66.00,119.00) | 0.135 |
| Globulin, g/L (IQR) | 23.60(19.80,27.20) | 23.10(19.20,27.10) | 0.262 |
| Triglyceride, mmol/L (IQR) | 1.27(0.88,1.94) | 1.30(0.91,1.85) | 0.787 |
| Total Cholesterol, mmol/L (IQR) | 3.25(2.55,4.05) | 3.08(2.32,3.85) | **0.001** |
| Comorbidities, n (%) |  |  |  |
| Hypertension | 300(37.3) | 271(33.7) | 0.145 |
| Diabetes mellitus | 199(24.7) | 201(25.0) | 0.954 |
| Hyperlipidemia | 122(15.2) | 100(12.4) | 0.129 |
| Other comorbidities | 233(28.9) | 234(29.1) | 1.000 |
| Medication, n (%) |  |  |  |
| Diuretics | 497(61.7) | 103(12.8) | **< 0.001** |
| ACEI/ARB | 3(0.4) | 1(0.1) | 0.625 |
| β-blockers | 33(4.1) | 128(15.9) | **< 0.001** |
| CCBs | 61(7.6) | 186(23.1) | **< 0.001** |
| NSAIDs | 60(7.5) | 195(24.2) | **< 0.001** |
| Types of PPIs, n (%) |  |  |  |
| Esomeprazole | — | 671(83.4) | — |
| Ilaprazole | — | 333(41.4) | — |
| Omeprazole | — | 27(3.4) | — |
| Pantoprazole | — | 51(6.3) | — |

**Abbreviation:** IQR, interquartile range; ACEI/ARB, Angiotensin-Converting Enzyme Inhibitor / Angiotensin Ⅱ Receptor Blocker; CCB, Calcium Channel Blockers; NSAID, Non-Steroidal Anti-Inflammatory Drugs; Other comorbidities, Coronary artery disease, Cerebrovascular accident; Heart failure, Chronic kidney disease, Chronic liver disease, Rheumatoid arthritis; PPI, Proton pump inhibitor.
